# Supplementary material for: A unified framework for inferring the multi-scale organization of chromatin domains from Hi-C
Source: PLoS Comput Biol. 2021 Mar 16;17(3):e1008834. doi: 10.1371/journal.pcbi.1008834 (PMC7997044; doi:10.1371/journal.pcbi.1008834)
Supplement: S1 Appendix — (PDF) [file pcbi.1008834.s001.pdf]

Supplementary Information for:

A unified framework for inferring the multi-scale organization of chromatin domains from Hi-C

Ji Hyun Bak, Min Hyeok Kim, Lei Liu, Changbong Hyeon

S1 Appendix

Gaussian polymer network for modeling chromosomes

Here we provide additional justifications for the use of harmonic potentials in the effective Hamiltonian, and consequently, a gaussian distribution for pairwise distances (**Eq 2**).

Even in the interphase that displays less amount of activity than mitotic phase, continuous events of free energy consumption break the detailed balance condition, driving the chromosome out of equilibrium [1–5]. However, chromosome dynamics in each phase during the cell cycle is slow enough [6–9] that the system remains in local mechanical equilibrium over an extended time period, as captured by the stable patterns in the Hi-C data [10]. Although the notion of cell-to-cell variation in a population of cells is appreciated in the literature [7, 9, 11–16], fluorescence measurement still indicates that the spatial distances between pairs of chromatin segments can be well described by the gaussian distribution [17–20] (**S1 Fig**). This motivates us to model the chromosome structure using a gaussian polymer network whose configuration fluctuates around a mechanically equilibrated local basin of attraction [10, 21–24].

The concept of an effective Hamiltonian consisting of harmonic potential terms is not new; it has been widely employed to study a variety of systems, including the phase transition of vulcanized macromolecules with increasing numbers of crosslinks [25, 26], and the fluctuation dynamics of native proteins (gaussian network model, [27]). Furthermore, a slightly modified, but essentially identical, form of Hamiltonian was used to study the dynamics of folding/unfolding transitions of a single RNA molecule under external force in the name of generalized Rouse model [28].

Whereas the success of the gaussian polymer network model does not necessarily guarantee its extension to the modeling of chromosomes, our use of a gaussian distribution for the pairwise distance between two segments in the polymer is empirically justified. The Gaussian-like pairwise distance distributions reported by fluorescence measurements of the chromosome (**S1 Fig**), and the agreement of the 3D structural properties inferred by modeling approaches [10, 23, 24] that share the same philosophy, with particular emphasis on our recent approach of *heterogeneous loop model* (HLM) [10, 29], suggest that Gaussian polymer networks provides a reasonable approximation of the energy landscape for the mixture of those subpopulations.

As a side note, it is worth highlighting the versatility of the Gaussian polymer network model in representing the complex topology of chromosome conformation. For the conventional Rouse chain whose monomers along the backbone are constrained by an energy hamiltonian $H = (k/2)\sum_i^{N-1}(r_{i+1} - r_i)^2$ with a uniform spring constant k, it is straightforward to show that $\langle r_{ij}^2 \rangle \sim |i - j|$. Furthermore, if two monomers are in close proximity to form a contact ($r_{ij} < r_c$), then one can obtain the contact probability between monomers i and j in the chain backbone as $p_{ij} = \int_0^{r_c} dr_{ij} P(r_{ij}) \sim |i - j|^{-3/2}$.

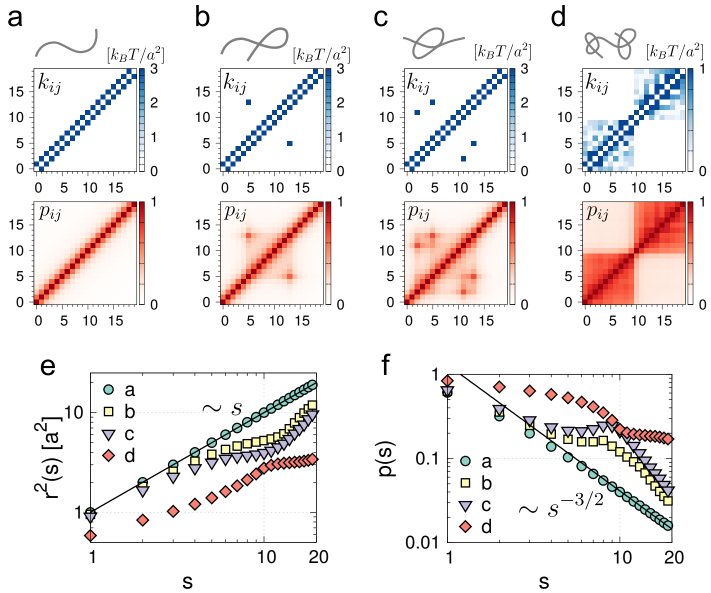

Fig A. Heterogenous loop model [10] to compare the contact probabilities of Gaussian polymer networks. (a-d) Four examples of polymer models composed of 20 monomers with different interaction strength matrix $[k_{ij}]$ (top row), and the corresponding contact probability matrices $[p_{ij}]$ (second row) calculated with $r_c = 1$. **(e)** the mean square distance and **(f)** the contact probability $p(s)$ are calculated as a function of the genomic distance, s, for the four different models (a-d). Scaling results in (e) and (f) show that even the Gaussian polymer network model can produce rich multi-scale structure with domains.

However, adding just a few non-nearest-neighbor interactions to the Rouse model makes the results highly nontrivial. To illustrate this, we explicitly compared the contact probability map of a linear Gaussian chain (Rouse chain), and those of Gaussian polymer network models with varying numbers of non-nearest-neighbor interactions, which were calculated from the HLM-generated structural ensemble [10] (**Fig A**). The statistical behavior of Gaussian polymer network model differs from that of the linear "Gaussian" chain. The mean square distance $\langle r_{ij}^2 \rangle$ no longer scales linearly with the separation $s \equiv |i - j|$ (**Fig A**), and the contact probability p_{ij} (or $p(s)$) is no longer described with a simple scaling relation (**Fig A**). The simple modification to the Rouse model, resulting in the Gaussian polymer network model, allows one to explore many different issues of chromosomes. In fact, our recent work based on HLM [10] demonstrated several case studies, substantiating the various experimental measurements on chromosome conformation by solving the inverse-problem of inferring chromosome structures from Hi-C data.

References

1. Bruinsma R, Grosberg AY, Rabin Y, Zidovska A. Chromatin hydrodynamics. Biophys J. 2014;106(9):1871–1881.

2. Battle C, Broedersz CP, Fakhri N, Geyer VF, Howard J, Schmidt CF, et al. Broken detailed balance at mesoscopic scales in active biological systems. Science. 2016;352:604–607.

3. Hwang W, Hyeon C. Quantifying the Heat Dissipation from Molecular Motor's Transport Properties in Nonequilibrium Steady States. J Phys Chem Lett. 2017;8:250–256.

4. Hwang W, Hyeon C. Energetic Costs, Precision, and Transport Efficiency of Molecular Motors. J Phys Chem Lett. 2018;9:513–520.

5. Fang X, Kruse K, Lu T, Wang J. Nonequilibrium physics in biology. Rev Mod Phys. 2019;91:045004.

6. Rosa A, Everaers R. Structure and Dynamics of Interphase Chromosomes. PLoS Comput Biol. 2008;4:e1000153.

7. Kang H, Yoon YG, Thirumalai D, Hyeon C. Confinement-induced glassy dynamics in a model for chromosome organization. Phys Rev Lett. 2015;115:198102.

8. Liu L, Shi G, Thirumalai D, Hyeon C. Chain organization of human interphase chromosome determines the spatiotemporal dynamics of chromatin loci. PLoS Comp Biol. 2018;14(12):e1006617.

9. Shi G, Liu L, Hyeon C, Thirumalai D. Interphase Human Chromosome Exhibits Out of Equilibrium Glassy Dynamics. Nat Commun. 2018;9:3161.

10. Liu L, Kim MH, Hyeon C. Heterogeneous Loop Model to Infer 3D Chromosome Structures from Hi-C. Biophys J. 2019;117:613–625.

11. Shi G, Thirumalai D. Conformational heterogeneity in human interphase chromosome organization reconciles the FISH and Hi-C paradox. Nature Commun. 2019;10(1):1–10.

12. Bintu B, Mateo LJ, Su JH, Sinnott-Armstrong NA, Parker M, Kinrot S, et al. Super-resolution chromatin tracing reveals domains and cooperative interactions in single cells. Science. 2018;362:eaau1783.

13. Nir G, Farabella I, Estrada CP, Ebeling CG, Beliveau BJ, Sasaki HM, et al. Walking along chromosomes with super-resolution imaging, contact maps, and integrative modeling. PLoS genetics. 2018;14(12):e1007872.

14. Nagano T, Lubling Y, Stevens TJ, Schoenfelder S, Yaffe E, Dean W, et al. Single-cell Hi-C reveals cell-to-cell variability in chromosome structure. Nature. 2013;502(7469):59–64.

15. Stevens TJ, Lando D, Basu S, Atkinson LP, Cao Y, Lee SF, et al. 3D structures of individual mammalian genomes studied by single-cell Hi-C. Nature. 2017;544(7648):59–64.

16. Finn EH, Pegoraro G, Brandão HB, Valton AL, Oomen ME, Dekker J, et al. Extensive heterogeneity and intrinsic variation in spatial genome organization. Cell. 2019;176(6):1502–1515.

17. Wang S, Su JH, Beliveau BJ, Bintu B, Moffitt JR, Wu Ct, et al. Spatial organization of chromatin domains and compartments in single chromosomes. Science. 2016;353(6299):598–602.

18. Szabo Q, Jost D, Chang JM, Cattoni DI, Papadopoulos GL, Bonev B, et al. TADs are 3D structural units of higher-order chromosome organization in Drosophila. Science advances. 2018;4(2):eaar8082.

19. Finn EH, Pegoraro G, Shachar S, Misteli T. Comparative analysis of 2D and 3D distance measurements to study spatial genome organization. Methods. 2017;123:47–55.

20. Giorgetti L, Galupa R, Nora EP, Piolot T, Lam F, Dekker J, et al. Predictive polymer modeling reveals coupled fluctuations in chromosome conformation and transcription. Cell. 2014;157(4):950–963.

21. Sauerwald N, Zhang S, Kingsford C, Bahar I. Chromosomal dynamics predicted by an elastic network model explains genome-wide accessibility and long-range couplings. Nucleic acids research. 2017;45(7):3663–3673.

22. Zhang S, Chen F, Bahar I. Differences in the intrinsic spatial dynamics of the chromatin contribute to cell differentiation. Nucleic Acids Research. 2020;48:1131–1145.

23. Le Treut G, Képès F, Orland H. A Polymer Model for the Quantitative Reconstruction of Chromosome Architecture from HiC and GAM Data. Biophys J. 2018;115(12):2286–2294.

24. Shinkai S, Nakagawa M, Sugawara T, Togashi Y, Ochiai H, Nakato R, et al. PHi-C: deciphering Hi-C data into polymer dynamics. NAR Genomics and Bioinfo. 2020;2(2):lqaa020.

25. Goldbart PM, Zippelius A. Amorphous solid state of vulcanized macromolecules: A variational approach. Phys Rev Lett. 1993;71:2256–2259.

26. Bryngelson JD, Thirumalai D. Internal Constraints Induce Localization in an Isolated Polymer Molecule. Phys Rev Lett. 1996;76:542–545.

27. Haliloglu T, Bahar I, Erman B. Gaussian Dynamics of Folded Proteins. Phys Rev Lett. 1997;79:3090–3093.

28. Hyeon C, Morrison G, Thirumalai D. Force-dependent hopping rates of RNA hairpins can be estimated from accurate measurement of the folding landscapes. Proc Natl Acad Sci U S A. 2008;105(28):9604–9609.

29. Liu L, Hyeon C. Revisiting the organization of Polycomb-repressed domains: 3D chromatin models from Hi-C compared with super-resolution imaging. Nucleic Acids Research. 2020; p. gkaa932.
