# Supplementary material for: A unified framework for inferring the multi-scale organization of chromatin domains from Hi-C
Source: PLoS Comput Biol. 2021 Mar 16;17(3):e1008834. doi: 10.1371/journal.pcbi.1008834 (PMC7997044; doi:10.1371/journal.pcbi.1008834)
Supplement: S2 Appendix — (PDF) [file pcbi.1008834.s002.pdf]

Supplementary Information for:

A unified framework for inferring the multi-scale organization of chromatin domains from Hi-C

Ji Hyun Bak, Min Hyeok Kim, Lei Liu, Changbong Hyeon

S2 Appendix

Derivation of the likelihood function

Here we derive the likelihood function, **Eq 14**.

Problem: We want to compute

$$p(\mathbf{x}|\mathbf{s},\mathbf{g}) = \left\langle \delta^N\left(\mathbf{x} - \mathbf{f}(\eta,\epsilon)\right)\right\rangle_{\eta,\epsilon} \tag{S2-1}$$

with the following assumptions:

- $\mathbf{x} \in \mathbb{R}^N$ is a sequence of normalized and uncorrelated observations, with zero mean $\langle \mathbf{x} \rangle = \mathbf{0}_N$ and unit covariance $\mathrm{Cov}(\mathbf{x}) = I_N$.
- $\mathbf{s} = (s_1, \cdots, s_N)$ is a clustering map that assigns each site $i \in \{1, \cdots, N\}$ to a cluster index $s_i \in \{1, \cdots, K\}$. Without loss of generality, we can assume that $s_i \leq s_j$ whenever $i < j$ (ordered indexing).
- $\eta \sim \mathcal{N}(\mathbf{0}_N, \Lambda)$ and $\epsilon \sim \mathcal{N}(\mathbf{0}_N, \Sigma)$ are i.i.d. gaussian random variables, where Λ and Σ are $N \times N$ covariance matrices. The cluster-dependent covariance is a block diagonal matrix $\Lambda = [\Lambda_s] = [\mathbf{1}_{n_s}\mathbf{1}_{n_s}^\top]$, defined element-wise as $(\Lambda)_{ij} = \delta_{s_i,s_j}$. The site-wise variation is assumed to be uncorrelated, with a unit covariance matrix $\Sigma = I_N$, or $(\Sigma)_{ij} = \delta_{ij}$.
- The clustering strength $\mathbf{g} = (g_1, \cdots, g_K)$ parameterizes the target function $\mathbf{f}$, defined element-wise as

$$f_i(\eta,\epsilon) = \frac{\sqrt{g_{s_i}}\eta_i + \epsilon_i}{\sqrt{1 + g_{s_i}}}, \quad i = 1, \cdots, N. \tag{S2-2}$$

Two lemmas will be useful. The Gaussian integral lemma:

$$\int_{\mathbb{R}^N} d\mathbf{z}\; \mathcal{N}(\mathbf{z}|\boldsymbol{\mu}, M)\; e^{i\mathbf{a}^\top \mathbf{z}} = \exp\left(-\frac{1}{2}\mathbf{a}^\top M \mathbf{a}\right), \quad \mathbf{a} \in \mathbb{R}^N; \tag{S2-3}$$

and the Sherman-Morrison formula:

$$(A + \mathbf{u}\mathbf{v}^\top)^{-1} = A^{-1} - \frac{A^{-1}\mathbf{u}\mathbf{v}^\top A^{-1}}{1 + \mathbf{v}^\top A^{-1}\mathbf{u}}. \tag{S2-4}$$

Solution: Let us abbreviate the coefficients as $\alpha_s \equiv \sqrt{g_s/(1+g_s)}$ and $\beta_s \equiv 1/\sqrt{1+g_s}$, such that $f_i = \alpha_{s_i}\eta_i + \beta_{s_i}\epsilon_i$. Further define $A \equiv \mathrm{diag}(\alpha_{s_i})$ and $B \equiv \mathrm{diag}(\beta_{s_i})$, to write $\mathbf{f} = A\boldsymbol{\eta} + B\boldsymbol{\epsilon}$. Taking the inverse Fourier transform of the Dirac delta function, we can write $\delta^N(\mathbf{x}-\mathbf{f}) = \int \frac{d\mathbf{k}}{(2\pi)^N} e^{i(\mathbf{x}-\mathbf{f})^\top \mathbf{k}} = \int \frac{d\mathbf{k}}{(2\pi)^N} e^{i(\mathbf{x}-A\boldsymbol{\eta}-B\boldsymbol{\epsilon})^\top \mathbf{k}}$, where $\int = \int_{\mathbb{R}^N}$ unless otherwise specified. Now we can rewrite **Eq S2-1**, and evaluate the gaussian integrals using the lemma (**Eq S2-3**):

$$\begin{aligned} p(\mathbf{x}|\mathbf{s},\mathbf{g}) &= \int \frac{d\mathbf{k}}{(2\pi)^N}\ e^{i\mathbf{x}^\top \mathbf{k}} \int d\boldsymbol{\eta}\ \mathcal{N}(\boldsymbol{\eta})\ e^{-iA\boldsymbol{\eta}^\top \mathbf{k}} \int d\boldsymbol{\epsilon}\ \mathcal{N}(\boldsymbol{\epsilon})\ e^{-iB\boldsymbol{\epsilon}^\top \mathbf{k}} \\ &= \int \frac{d\mathbf{k}}{(2\pi)^N}\ \exp\left(i\mathbf{x}^\top\mathbf{k} - \frac{1}{2}(A\mathbf{k})^\top\Lambda(A\mathbf{k}) - \frac{1}{2}(B\mathbf{k})^\top\Sigma(B\mathbf{k})\right) \\ &= \int \frac{d\mathbf{k}}{(2\pi)^N}\ \exp\left(i\mathbf{x}^\top\mathbf{k} - \frac{1}{2}\mathbf{k}^\top Q\mathbf{k}\right), \end{aligned} \tag{S2-5}$$

where $Q \equiv (A\Lambda A + B\Sigma B)$. Recognizing that this is another (unnormalized) gaussian integral with covariance matrix Q^{-1}, we use the lemma (**Eq S2-3**) once again:

$$\begin{aligned} p(\mathbf{x}|\mathbf{s},\mathbf{g}) &= \sqrt{(2\pi)^N \det Q^{-1}} \int \frac{d\mathbf{k}}{(2\pi)^N}\ \mathcal{N}(\mathbf{k}|\mathbf{0}, Q^{-1})\ e^{i\mathbf{x}^\top\mathbf{k}} \\ &= \exp\left(-\frac{1}{2}\mathbf{x}^\top Q^{-1}\mathbf{x} - \frac{1}{2}\log\det Q\right). \end{aligned} \tag{S2-6}$$

With uncorrelated $\boldsymbol{\epsilon}$, both Q and Q^{-1} are block diagonal matrices, the exponent is completely separable by clusters:

$$\log p(\mathbf{x}|\mathbf{s},\mathbf{g}) = -\frac{1}{2}\sum_{s=1}^{K}\left(\mathbf{x}_s^\top Q_s^{-1}\mathbf{x}_s + \log\det Q_s\right), \tag{S2-7}$$

where $\mathbf{x}_s$ is the corresponding n_s-dimensional subset of $\mathbf{x}$, and $Q_s = A_s\Lambda_s A_s + B_s\Sigma_s B_s = \alpha_s^2\Lambda_s + \beta_s^2\Sigma_s$, is the $n_s \times n_s$ block matrix corresponding to cluster index s; element-wise, $(Q_s)_{ij} = \alpha_s^2 + \beta_s^2\delta_{ij}$.

We now simplify the two terms in the summand of **Eq S2-7**, and show that the resulting expression corresponds to **Eq 14**. First, the quadratic term can be expanded by using the Sherman-Morrison formula (**Eq S2-4**):

$$Q_s^{-1} = (\beta_s^2 I_{n_s} + (\alpha_s\mathbf{1}_{n_s})(\alpha_s\mathbf{1}_{n_s})^\top)^{-1} = \frac{1}{\beta_s^2}\left(I - \frac{(\alpha_s^2/\beta_s^2)\mathbf{1}\mathbf{1}^\top}{1+(\alpha_s^2/\beta_s^2)\mathbf{1}^\top\mathbf{1}}\right). \tag{S2-8}$$

The quadratic form is

$$\mathbf{x}_s^\top Q_s^{-1}\mathbf{x}_s = (1+g_s)\left(n_s - \frac{g_s c_s}{1+g_s n_s}\right), \tag{S2-9}$$

where $\mathbf{x}_s^\top\mathbf{x}_s = \sum_{i=1}^N (x_i)^2\delta_{s_i,s} \approx \langle x_i^2\rangle \sum_{i=1}^N \delta_{s_i,s} = n_s$, and $\mathbf{x}_s^\top(\mathbf{1}\mathbf{1}^\top)\mathbf{x}_s = \sum_{i,j=1}^N x_i x_j \delta_{s_i,s}\delta_{s_j,s} \equiv c_s$.

Second, the log-determinant term can be calculated by considering the eigenvalues of the matrix Q_s. Solving for $Q_s\mathbf{z} = \lambda_s\mathbf{z}$ for an arbitrary n_s-dimensional vector $\mathbf{z}$,

$$\lambda_s\mathbf{z} = \alpha_s^s(\mathbf{1}^\top\mathbf{z})\mathbf{1} + \beta_s^2\mathbf{z}; \tag{S2-10}$$

there are two types of solutions. The first possibility is to have the eigenvector $\mathbf{z} \propto \mathbf{1}$, in which case $\lambda_{s,1} = \alpha_s^2 n_s + \beta_s^2 = (1+g_s n_s)/(1+g_s)$. The other possibility is to have

$(\lambda_s - \beta_s^2)\mathbf{z}$ vanish, where $\lambda_{s,2} = \cdots = \lambda_{s,n_s} = \beta_s^2 = 1/(1+g_s)$; the degenerate eigenvectors span the remaining $(n_s - 1)$-dimensional subspace. Therefore

$$\det(Q_s) = (\alpha_s^2 n_s + \beta_s^2) \cdot (\beta_s^2)^{n_s - 1} = \frac{1 + g_s n_s}{(1+g_s)^{n_s}}, \tag{S2-11}$$

and

$$\log\det(Q_s) = \log(1 + g_s n_s) - n_s \log(1 + g_s). \tag{S2-12}$$

Substitution of **Eq S2-9** and **Eq S2-12** into **Eq S2-7** yields **Eq 14**.
