# Supplementary material for: A unified framework for inferring the multi-scale organization of chromatin domains from Hi-C
Source: PLoS Comput Biol. 2021 Mar 16;17(3):e1008834. doi: 10.1371/journal.pcbi.1008834 (PMC7997044; doi:10.1371/journal.pcbi.1008834)
Supplement: S3 Appendix — (PDF) [file pcbi.1008834.s003.pdf]

Supplementary Information for:

A unified framework for inferring the multi-scale organization of chromatin domains from Hi-C

Ji Hyun Bak, Min Hyeok Kim, Lei Liu, Changbong Hyeon

S3 Appendix

The inference algorithm

Sampling Markov chain Monte Carlo (MCMC) sampling was employed to find the minimum value of the total cost function $\mathcal{H}$. At each trial move from the current state $\mathbf{s}$ to the next state $\mathbf{s}'$, the move is accepted with a probability $\min(1, \alpha)$, where $\alpha(\mathbf{s}, \mathbf{s}') = \exp\left[-(\mathcal{H}(\mathbf{s}'|\mathbf{C}) - \mathcal{H}(\mathbf{s}|\mathbf{C}))/T\right]$. In sampling the space of CD solutions, a move from a state $\mathbf{s}$ to another state $\mathbf{s}'$ is defined such that the two CD solutions $(\mathbf{s}, \mathbf{s}')$ differ only by one genomic segment. More precisely, because a CD solution is invariant upon permutations of the domain indices, the distance between $\mathbf{s}$ and $\mathbf{s}'$ is uniquely defined as the *minimal* number of mismatches over all possible domain index permutations.

To ensure that the sampling is properly conducted, we continue the sampling until each chain collects $t_{\text{tot}} \geq 5\tau^*$ samples in the CD solution space. The "relaxation time" τ^* is defined as the number of steps at which the autocorrelation function $R(\tau)$, drops significantly ($< 1/e$). The autocorrelation function is calculated as

$$R(\tau) = \frac{1}{\sigma^2} \left\langle (\mathcal{H}(\mathbf{s}_t|\mathbf{C}) - \mu)(\mathcal{H}(\mathbf{s}_{t+\tau}|\mathbf{C}) - \mu) \right\rangle_t, \tag{S3-1}$$

where $\mathbf{s}_t$ is the t-th sample in the chain, and μ and σ are the mean and standard deviation of $\mathcal{H}$. The average $\langle \cdot \rangle_t$ is taken over all pairs of samples with a delay of τ.

Simulated annealing The simulated annealing process is described below. Also see **Fig A** for an example of simulated annealing in our Multi-CD algorithm.

Initialization. An initial configuration $\mathbf{s}^{(0)}$ is generated in two random steps. First, the total number of CDs, K, is drawn randomly from the set of integers $\{1, \cdots, N\}$. Then, each genomic segment $i \in \{1, \cdots, N\}$ is allocated randomly into one of the CDs, $k \in \{1, 2, \cdots, K\}$. The initial temperature T_0 is determined such that the acceptance probability for the "worst" move around $\mathbf{s}^{(0)}$ is 0.5.

Iteration. At each step r, the temperature is fixed at T_r. We sample the target distribution $p_r(\mathbf{s}|\mathbf{C}) \propto \exp(-\mathcal{H}(\mathbf{s}|\mathbf{C})/T_r)$, using the Metropolis-Hastings sampler described above. For the next step $r+1$, the temperature is lowered by a constant cooling factor $c_{\text{cool}} \in (0, 1)$, such that the next temperature is $T_{r+1} = c_{\text{cool}} \cdot T_r$. We used $c_{\text{cool}} = 0.95$ in this study.

Final solution. The annealing is repeated until the temperature reaches T_f. We used $T_f = 0.03$. Then we quench the system to the closest local minimum by performing gradient descent. Because there is still no guarantee that the global minimum is found, we tried a batch of at least 10 different initial configurations and chose the final state $\mathbf{s}^*$ that gives the minimal $\mathcal{H}(\mathbf{s}^*|\mathbf{C})$.

Robustness of solutions over data subset choices The domain solutions reported by Multi-CD are robust over different choices as to which subsets of Hi-C data we solve from. We showed that Multi-CD is practically *locality-preserving*, in the following sense. Suppose that $S_1, S_2 \subset \{1, 2, \cdots, N\}$ be two subsets (specifically, consecutive intervals) of the genomic range, and both include the two genomic segments i, j. At a given λ, if the pair (i, j) belongs to the same domain according to a domain solution based on the subset of data $\mathbf{C}_{S_1}$, most of the times it also belongs to the same domain when solved for the other subset $\mathbf{C}_{S_2}$. Also see **Fig B** for an example from the real data.

Computational cost Repeated sampling in the simulated annealing is the computational bottleneck for the current method. Whereas our final choice of parameters for the simulated annealing was on the conservative side, to prioritize accurate solutions over speed, it is often useful to adjust the parameters to enable lighter runs, especially for pilot studies. For the MCMC sampling at each fixed temperature, the chain length (set adaptively by the stopping condition; we used $5\tau^*$ throughout this study) could be reduced, for example to $3\tau^*$. In general, one can trade off the number of independent simulated annealing runs (try a larger number of initial configurations), which is readily parallelized, for a shorter sampling per run. For the simulated annealing, the temperature schedule can be accelerated by adjusting the cooling rate c_{cool} (currently 0.95); a smaller c_{cool}, such as 0.9, results in a faster annealing.

In addition to adjusting the simulated annealing parameters listed above, one can also use smaller data subsets (with smaller subset size N) for faster test runs. More specifically, we used a smaller data subset and adjusted simulated annealing parameters (shorter chain length, accelerated cooling rate, etc.) to perform pilot runs to determine a rough range of λ values that is meaningful for the given data. Then we performed a more thorough run to obtain the actual results. The full set of parameters that we used for the main analysis, as well as for the shorter test runs, can be found in our public code repository.

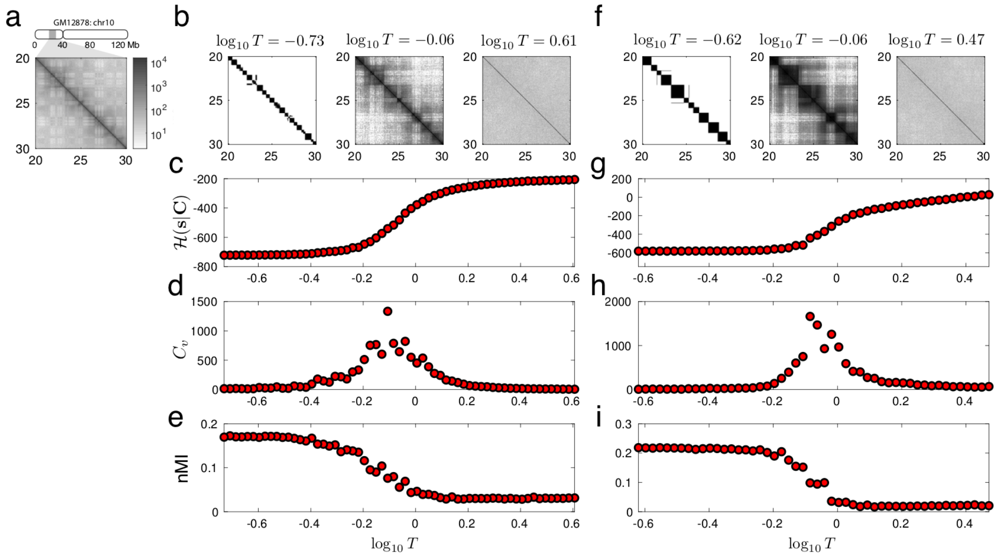

Fig A. Finding the best domain solution through simulated annealing. (a) A subset of Hi-C data, covering 10-Mb genomic region on chr10 of GM12878. **(b)** CD solutions, obtained from the Hi-C data in **(a)**, at three values of T for $\lambda = 0$. The CD solution at each T was constructed by $2,000$ sample trajectories being equilibrated. **(c-e)** We plot three quantities over varying T, where the simulated annealing from high to low T (right to left in figure) was used as a sampling protocol. **(c)** The effective energy hamiltonian $\mathcal{H}(\mathbf{s}|\mathbf{C})$. **(d)** The heat capacity $C_v = \langle \delta\mathcal{H}^2 \rangle / T^2$. **(e)** The normalized mutual information (nMI) between the domain solution and Hi-C matrix ($\log_{10} \mathbf{M}$). **(f-i)** Same analyses repeated for $\lambda = 10$.

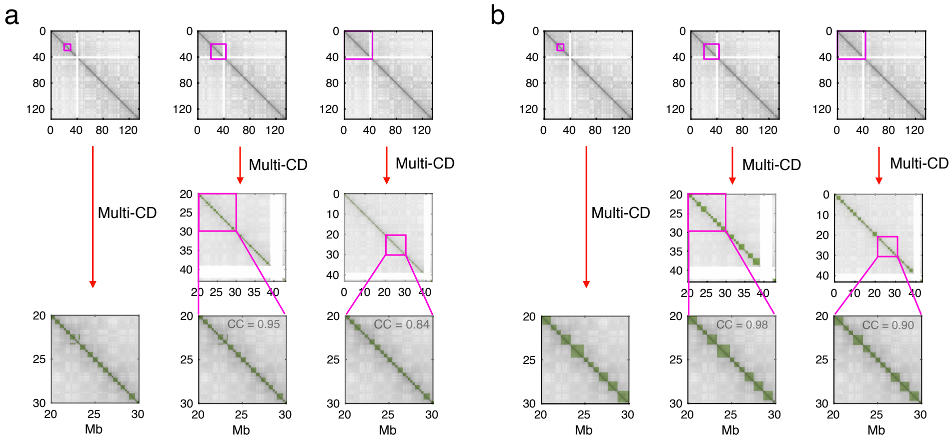

Fig B. Robustness of clustering solutions over different subsets of Hi-C data. The Hi-C data demarcated by the purple squares on the top panels are the input data used for Multi-CD analysis. The three panels from left to right on the bottom are the domain solutions from 10-Mb, 20-Mb, and 40-Mb Hi-C inputs. **(a)** For $\lambda = 0$, the correlation coefficients of 20-Mb Hi-C and 40-Mb Hi-C generated domain solutions with respect to the 10-Mb Hi-C generated one is 0.95 and 0.84, respectively. **(b)** Same calculations were carried out for λ=10.
