# Supplementary figures and images for: A unified framework for inferring the multi-scale organization of chromatin domains from Hi-C
Source: PLoS Comput Biol. 2021 Mar 16;17(3):e1008834. doi: 10.1371/journal.pcbi.1008834 (PMC7997044; doi:10.1371/journal.pcbi.1008834)

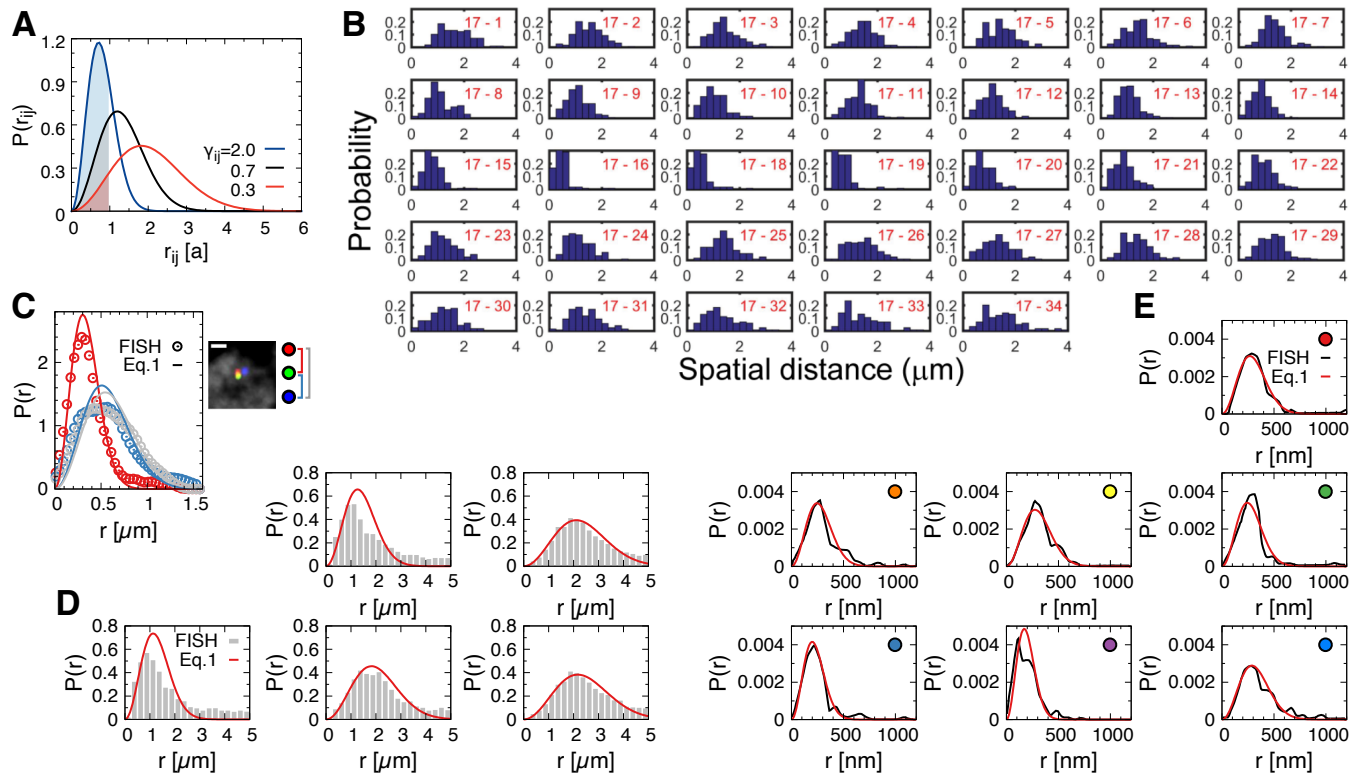

Supplement: S1 Fig — (A) Gaussian probability distribution plotting P(rij) with different values of γij (Eq 2). The shaded area in different colors represents the corresponding values of contact probabilities, (Eq 5 at rc = 1) (B) Distance distributions between one TAD (TAD17) and other TADs on Chr21 in human IMR90 cells measured with FISH. This figure was adapted from Fig 3A in [21]. (C) Distance distributions between three FISH probes on the X chromosome of male Drosophila embryos. The experimental data were digitized from Fig 3B in [42]. Their best fits to Eq 2 are plotted with solid lines. (D) Distance distributions between five pairs of FISH probes on chr1 in fibroblast cells. The experimental data (histograms) were digitized from Fig 4B in [43]. The fits using Eq 2 are plotted with solid lines. (E) Distance distributions between seven pairs of FISH probes in the Tsix/Xist region on the X chromosome of mouse ESC. The experimental data (black lines) were digitized from Fig 2F in [44], and their corresponding fits are shown in red. (PDF) [file pcbi.1008834.s004.pdf]

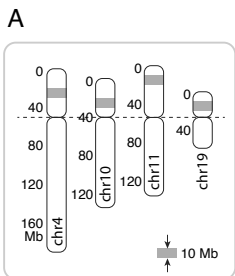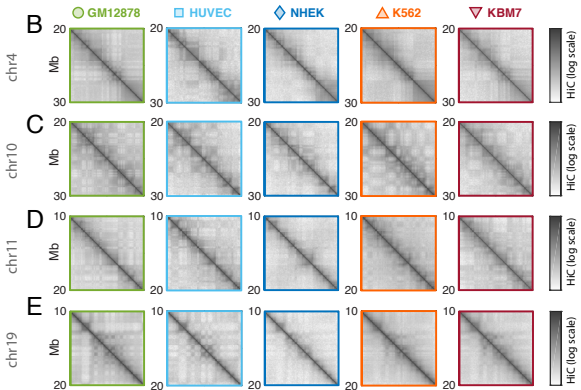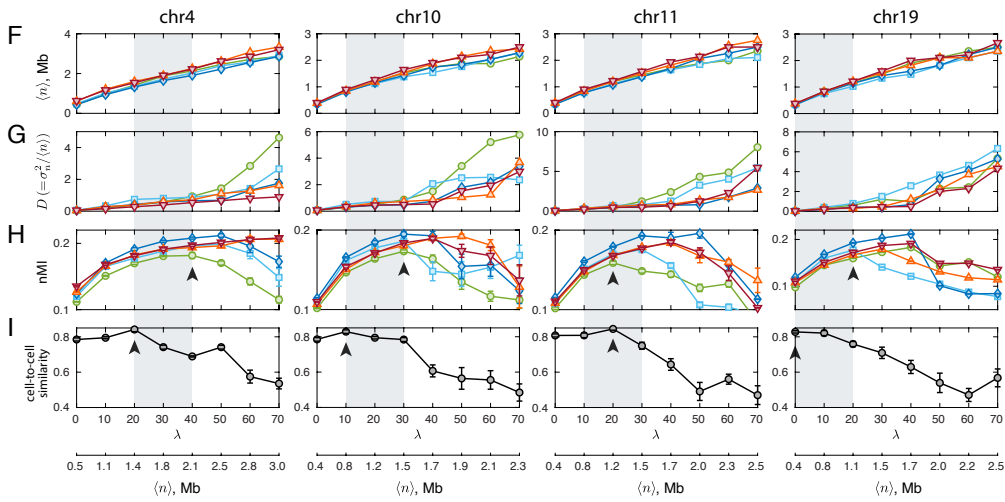

Supplement: S2 Fig — Extension of Fig 3. (A) Relative sizes of chromosomes considered, aligned at the centromeres. The gray shade in each chromosome indicates the 10-Mb interval for which we show the Hi-C data in the next panels. (B-E) Hi-C data for the corresponding 10-Mb genomic intervals of (B) chr4, (C) chr10, (D) chr11, and (E) chr19, for the five different cell lines respectively. All the panels for chr10 are reprints of Fig 3 in the main text. (F-I) Statistics of the domain solutions for chr4, chr10, chr11, and chr19. The five cell lines are color coded as indicated at the top of (B). (F) Mean domain size 〈n〉 as a function of λ. (G) The index of dispersion D(=σn2/〈n〉) of domain sizes. (H) The goodness of domain solutions, measured in terms of the normalized mutual information with respect to Hi-C data (log10 M). (I) The similarity of domain solutions across the five different cell types, measured by the Pearson correlation between binarized contact matrices. For each chromosome, arrows indicate the likely TAD scale (highest cell-to-cell similarity) and the likely meta-TAD scale (where the nMI is high and the index of dispersion D starts to diverge). (PDF) [file pcbi.1008834.s005.pdf]

**A**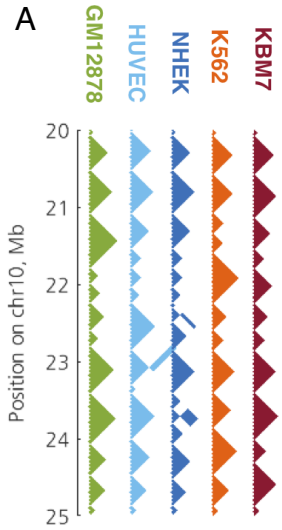**B**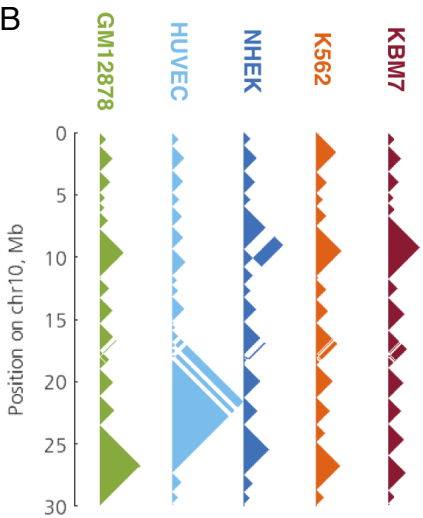

Supplement: S3 Fig — Extension of Fig 3. Shown are the domain solutions obtained from Multi-CD for the five different cell lines (GM12878, HUVEC, NHEK, K562, KBM7), at (A) λ = 0 and (B) λ = 40. (PDF) [file pcbi.1008834.s006.pdf]

$\langle n \rangle, \text{Mb}$

GM12878

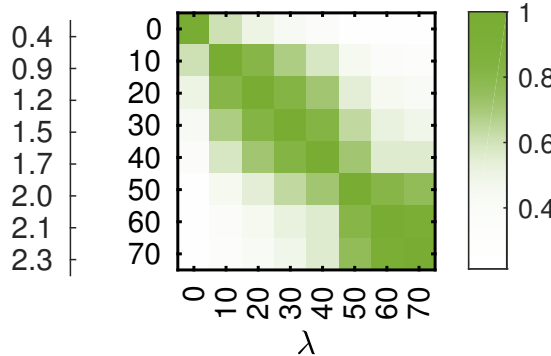

HUVEC

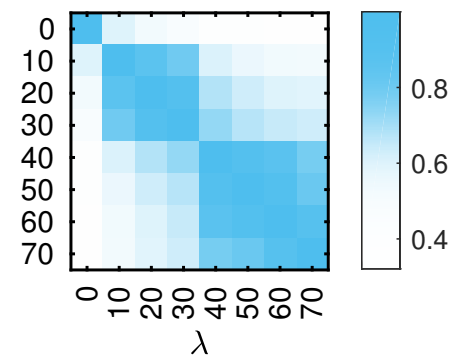

NHEK

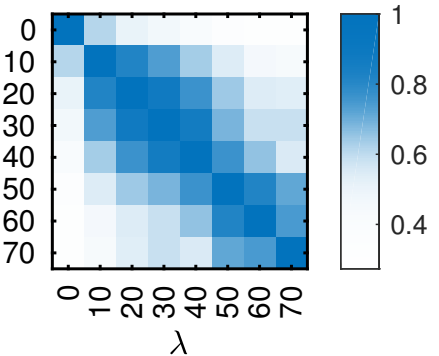

K562

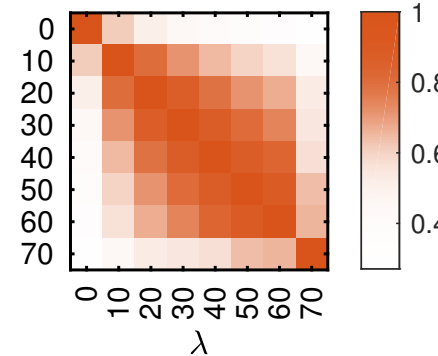

KBM7

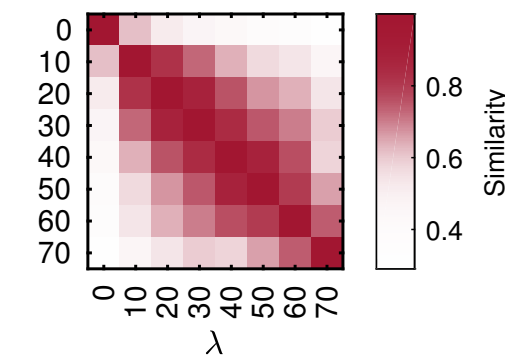

Similarity

Supplement: S4 Fig — Extension of Fig 3. We calculate the similarity between domain solutions at different λ in terms of Pearson correlation. The calculation was performed for chromosome 10 from five different cell lines. (PDF) [file pcbi.1008834.s007.pdf]

APBB1IP

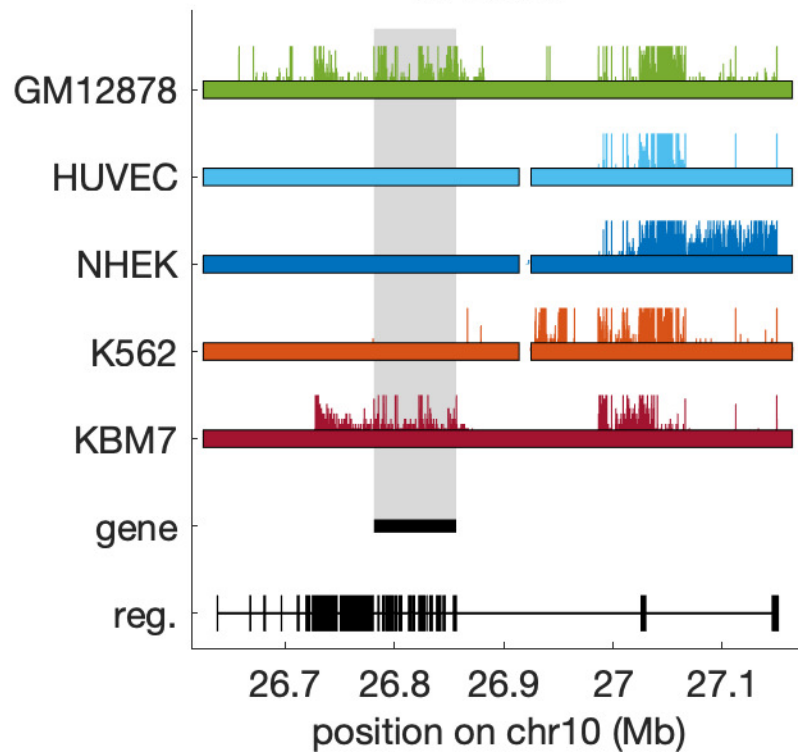

SVIL

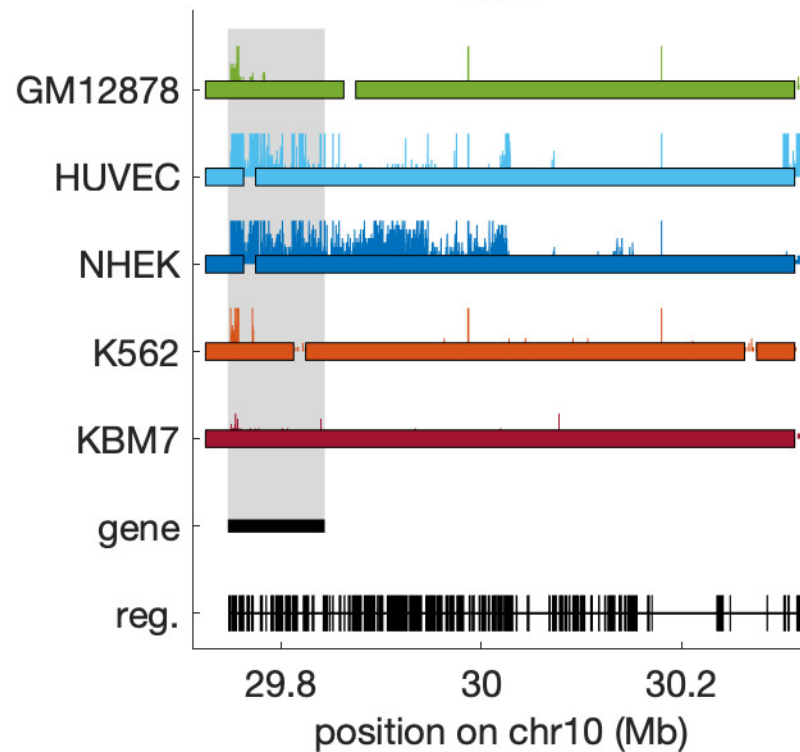

TSPAN15

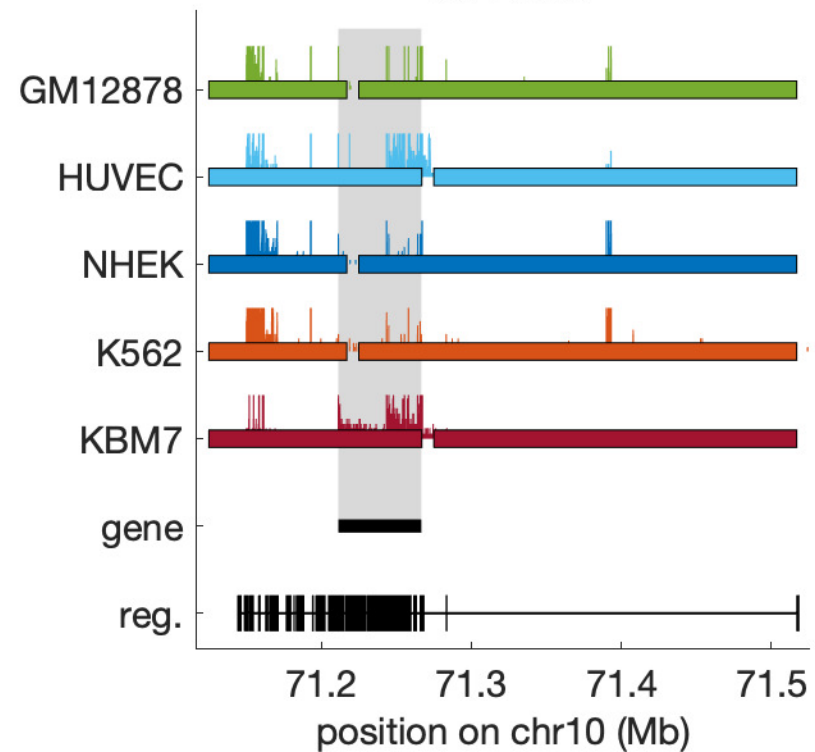

Supplement: S5 Fig — Extension of Fig 3K (gene APBB1IP, reproduced in the left panel), with two additional genes SVIL (middle panel) and TSPAN15 (right panel). Top five rows: Colored blocks indicate the TAD solutions for the five cell lines, as identified from our method. Colored hairy lines show the RNA-seq signal for the respective cell lines. Bottom two rows: Black horizontal bar and the gray shade mark the known position range of the gene. Finally, positions of all known regulatory elements for the gene are shown, as annotated in the GeneHancer database [61]. (PDF) [file pcbi.1008834.s008.pdf]

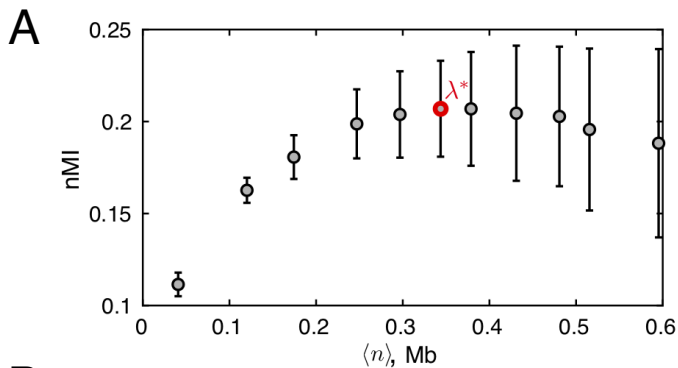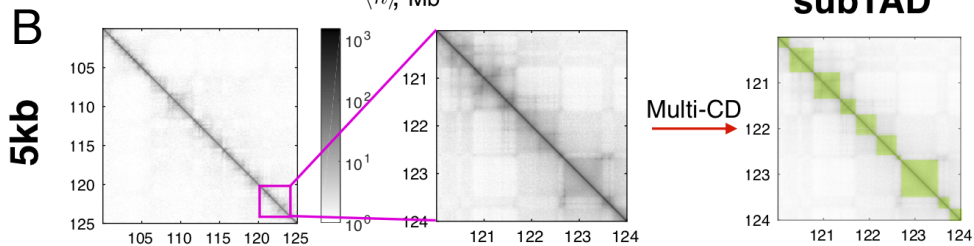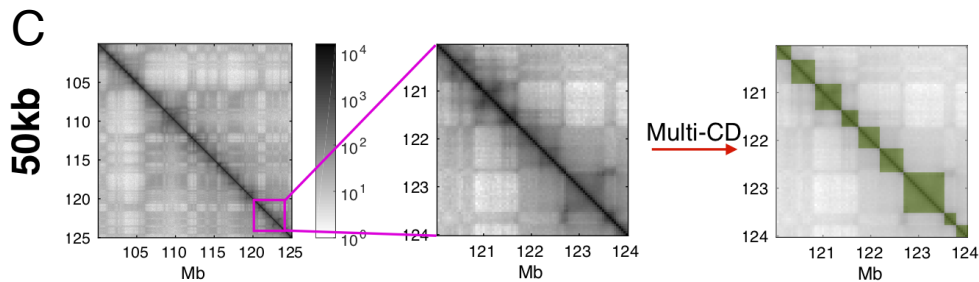

Supplement: S6 Fig — (A) The optimum cluster size, best describing 5-kb resolution Hi-C map in terms of nMI, is determined at 〈n〉 = 0.35 Mb, which is consistent with the sub-TAD size determined from 50-kb resolution Hi-C at λ = 0. (B-C) Comparison between Multi-CD solutions at different resolutions of the input Hi-C data, that point to the robustness of sub-TAD boundaries regardless of Hi-C resolution. (B) The best CD solution (corresponding to λ = λ* in panel (A)) for the 5-kb resolution Hi-C data in the 120-124 Mb region of the genome. (C) Solution for the same genomic interval from 50-kb Hi-C, determined at λ = 0. The two CD solutions are effectively identical, which supports our interpretation of sub-TAD as the unit of hierarchical chromosome organization. (PDF) [file pcbi.1008834.s009.pdf]

A

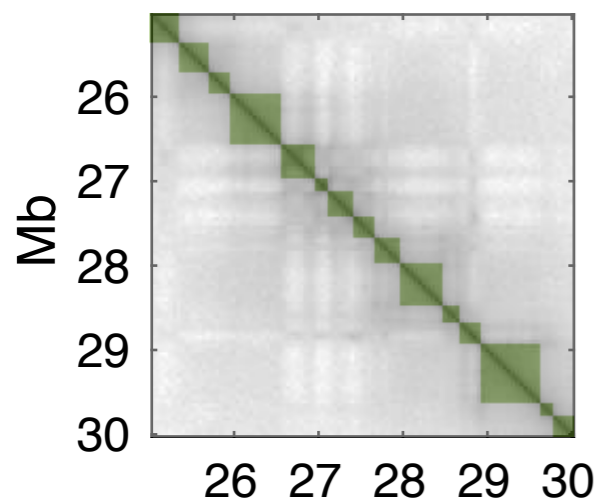

Multi-CD

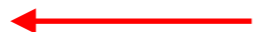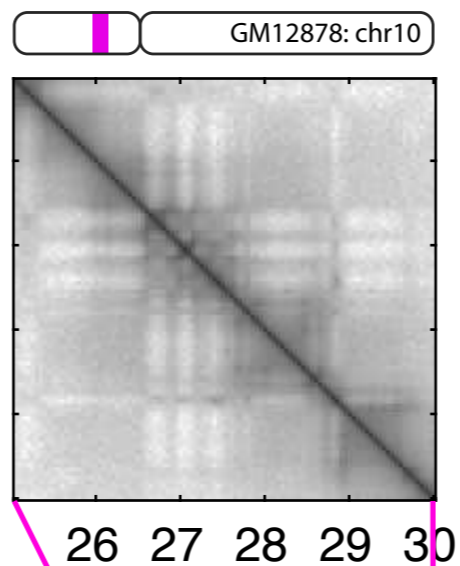

Arrowhead

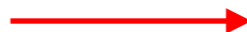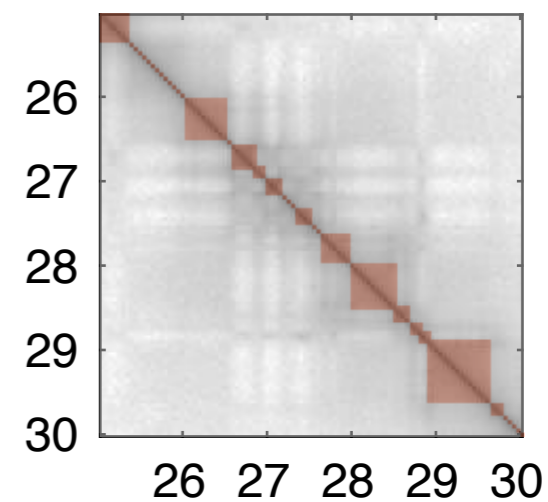

sub-TAD

B

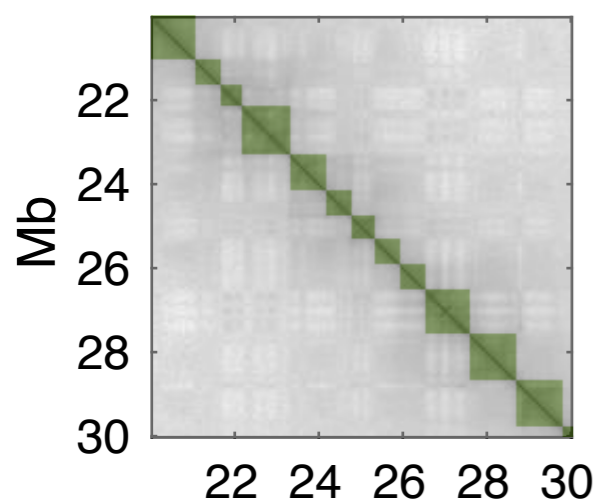

Multi-CD

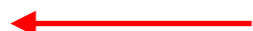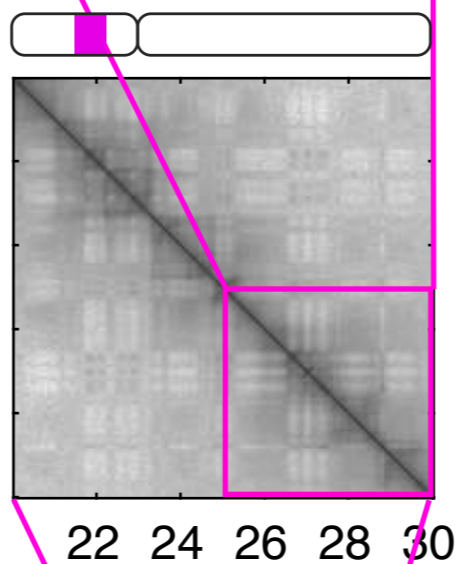

DomainCaller

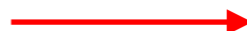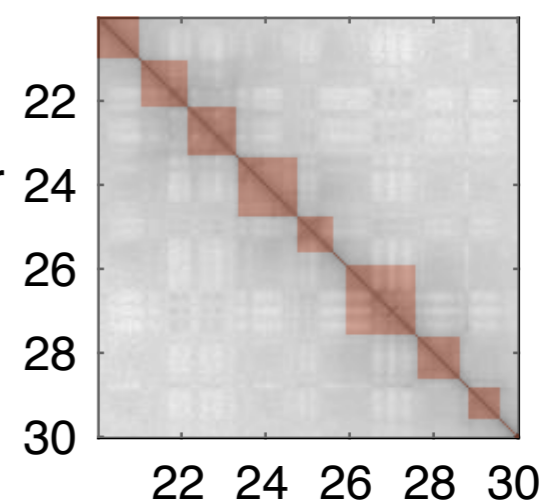

TAD

C

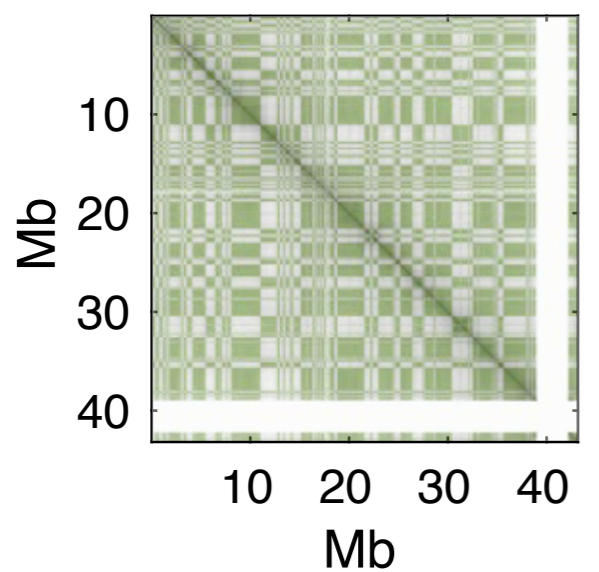

Multi-CD

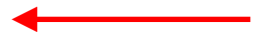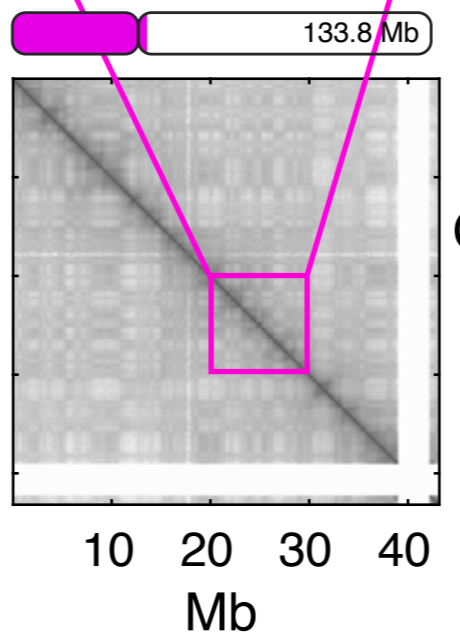

GaussianHMM

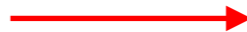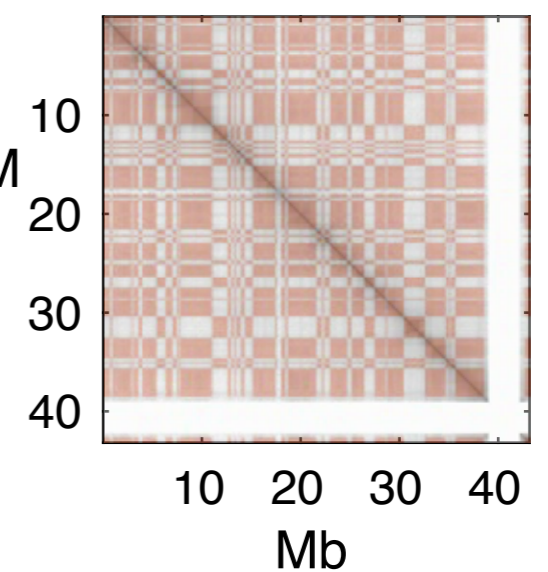

Compartment

Supplement: S7 Fig — Comparison between domain solutions obtained by three popular algorithms (ArrowHead, DomainCaller, GaussianHMM) (right column) and those by Multi-CD (left column), applied to 50-kb resolution Hi-C data. Three subsets from the same Hi-C data (log10 M), with different magnification (5, 10, and 40 Mb from top to bottom), are given in the middle column. ArrowHead algorithm [19] was used for identifying the domain structures of sub-TADs, DomainCaller [22] for TADs, and Gaussian Hidden Markov Model (GaussianHMM) [19] for compartments. Multi-CD use λ = 0, 10, 90, as the parameter values for identifying sub-TADs, TADs, and compartments, respectively. (PDF) [file pcbi.1008834.s010.pdf]

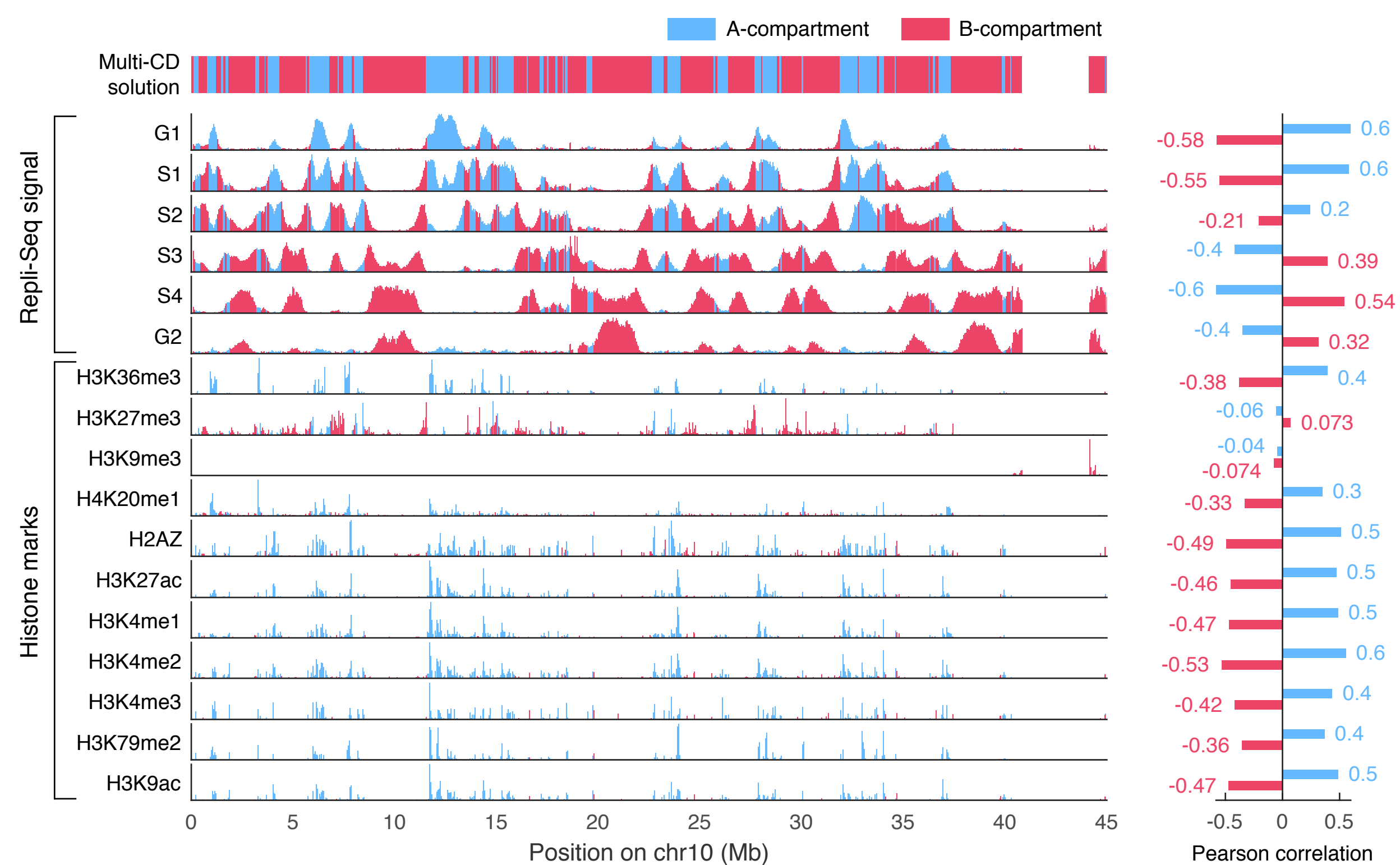

Supplement: S8 Fig — Extension of Fig 6C and 6D, which make comparison between the CD solutions for A/B-compartments by Multi-CD and epigenetic marks. The upper part with Repli-Seq signals is a reprint from the main text figure. The lower part shows histone marks on the corresponding genomic range. Majority of the histone marks are correlated with the A-compartment. The values of Pearson correlation between Repli-Seq signal or histone marks and A/B-compartment are given on the right. (PDF) [file pcbi.1008834.s011.pdf]

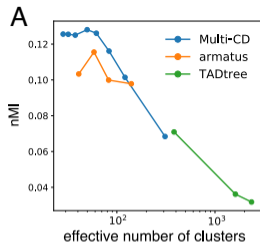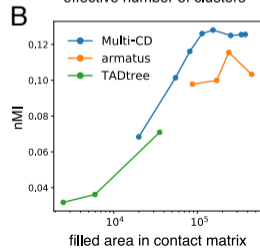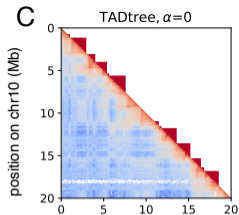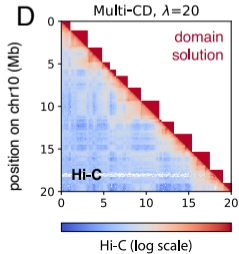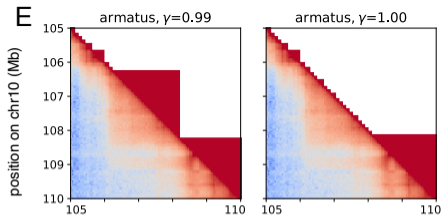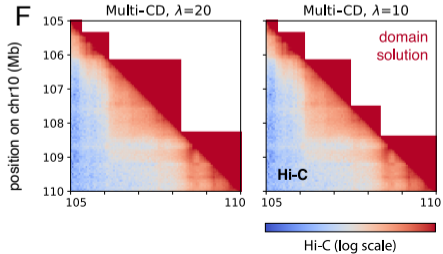

Supplement: S9 Fig — (A, B) Normalized mutual information between domain solutions at multiple scales, from Multi-CD, Armatus [34] and TADtree [33] respectively, and the log10 of KR-normalized Hi-C matrix for chr10 of the cell line GM12878. The scale of a domain solution s is measured in two ways, in terms of (A) the effective number of clusters, K(s)=exp(−∑k=1K(nk/N)log(nk/N)), where nk=∑i=1Nδsi,k is the domain size; and (B) the total area of 1’s in the corresponding binary contact matrix, (area)=∑i,j=1NBij where Bij=δsi,sj. All domain solutions from TADtree and Armatus were obtained using the respective default parameter settings. (C-F) Visual comparison of domains found by (C, D) TADtree and Multi-CD, and (E, F) Armatus and Multi-CD, at matching scales in terms of the average domain size. Domain solutions are shown in the upper triangle, colored by red (intra-domain) and white (extra-domain) for effective visualization. The lower triangle plots the corresponding subset of the Hi-C data (KR-normalized and in log10). Refer to the original papers [33, 34] for the definitions of the respective control parameters α (TADtree) and γ (Armatus). (PDF) [file pcbi.1008834.s012.pdf]
