# Supplementary material for: A unified framework for inferring the multi-scale organization of chromatin domains from Hi-C
Source: PLoS Comput Biol. 2021 Mar 16;17(3):e1008834. doi: 10.1371/journal.pcbi.1008834 (PMC7997044; doi:10.1371/journal.pcbi.1008834)
Supplement: Attachment — Submitted filename: PLOS_HiC_reply_letter.pdf [file pcbi.1008834.s013.pdf]

Response to Reviewers

Below we provide detailed point-by-point responses to the reviewers' comments. We present the original reviewer comments in **black** and our responses in **blue**.

Reviewer #1

The authors developed a unified method for analyzing Hi-C data and determine multi-scale organization of chromatin chain by changing the parameter λ. The method was applied to several cell lines and obtained valuable results that are consistent with the experimental observations. The methods and the results are quite interesting. I believe it is suitable for publication after the revision. There are some issues need to be addressed:

Thank you for the positive evaluation of our work! We will provide point-to-point responses to your comments below.

R1-1. The proposed method and the model are fully depended on the probability, which is obtained from the normalized Hi-C data. There are several normalization methods for Hi-C data, including ICE, KR, VC, etc. The authors should talk about the influence of the normalization algorithm on the results of the Sub-TADs, TADs, meta-TADs, and the compartments.

Indeed, our method relies on an appropriate translation of the raw Hi-C counts into a pairwise contact probability matrix, which is conventionally known as the *normalization* of the Hi-C data. Hi-C reports the frequency of observing a co-ligation between each pair of genomic loci, but the frequency of this observation depends on the combination of two independent probabilities: the pairwise contact probability between the two sites (the p_{ij} matrix in our model), and the non-uniform accessibility/observability of the individual sites (called the "one-dimensional biases" by [Rao et al., 2014]). The goal of normalization is to remove these single-site biases and only keep the pairwise contact contributions. Different normalization algorithms use different assumptions to achieve this common goal, and therefore the resulting matrices have slightly different statistical properties.

For our proposed method, we use the Knight-Ruiz (KR) algorithm [Knight & Ruiz, 2013] that explicitly specifies that the resulting matrix is doubly stochastic (i.e., behaves like a pairwise probability matrix, in the sense that each column/row sums to 1), which is suitable for our model that requires a contact probability.

That said, other, simpler normalization methods could give qualitatively similar results as well. For example, we were able to confirm that the main observations of our study remains robust when the square-root vanilla coverage (SQRTVC) normalization is used, as suggested by [Rao et al., 2014], which is a corrected version of the vanilla coverage (VC) normalization [Lieberman-Aiden et al., 2009]. See Fig R1(a, b) below.

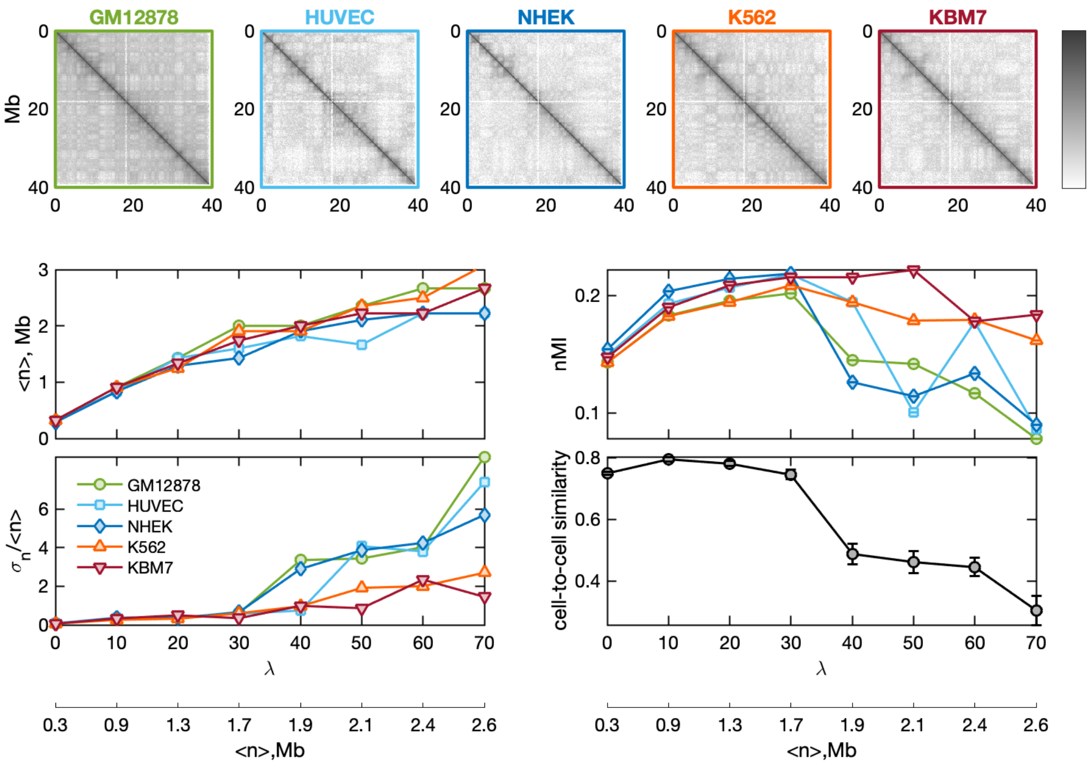

Fig R1a. TAD-like domain solutions, obtained from SQRTVC-normalized Hi-C data from [Rao et al., 2014]. Figure layout resembles Fig 3(a,d,e-h) in the manuscript. As in the KR-normalized results, the cell-to-cell conservation is strongest at λ=10 (TAD), and the nMI is consistently highest at λ=30 (meta-TAD); also, the two leukemia cells (K562 and KBM7) show different trends from the three normal cells (GM12878, HUVEC and NHEK).

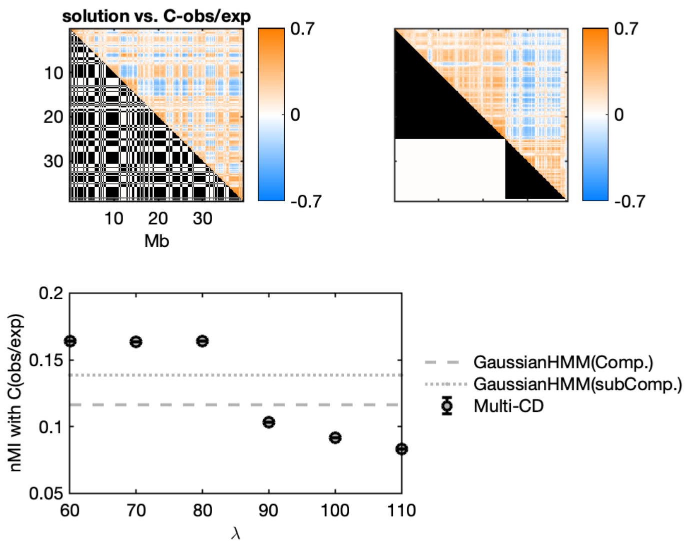

Fig R1b. Compartment-like (secondary) domain solutions from SQRTVC-normalized data from [Rao et al., 2014]. Figure layout resembles Fig 4(b-c,e). The compartment solution in the top row is obtained at λ=80, where the normalized mutual information (nMI) has a peak. (Note that the λ=80 solution from the KR-normalized data also essentially corresponds to the nMI peak; see our response to R1-2 below).
Error bars are not shown in this nMI plot, because this test was only performed for the first 40Mb-subset of chr10 (whereas in the main analysis we used three ~40Mb subsets to cover the entire chr10; see our response to R1-4 below).

R1-2. The authors claimed that the CD solutions correspond to the compartments at about λ=90. How to determine this value? If λ changes to 80 or 100, the distribution of the compartments stays unchanged or changes significantly? In addition, the results of large λ values (70 to 100) are not shown in Fig. 3.

We calculated the goodness of the domain solution at each λ at capturing the correlation pattern in the Hi-C data, in terms of the normalized mutual information (nMI; shown in Fig 4e in the manuscript, also reproduced in Fig R2 below) between the contact matrix of the inferred domain solution and the correlation pattern in the Hi-C. As shown in Fig 4e, λ=90 is where this nMI is maximized. However, the flat shape of the peak in the graph means that the compartment-like solution is not very sensitive to small changes in λ around the optimal value of 90. Indeed, when λ changes to 80 or 100 as the reviewer asked, we still get a very similar compartment solution (shown below in Fig R2). All three pairwise similarities between the three solutions at λ=80, 90 and 100, calculated as the Pearson correlation between the corresponding binarized contact matrices, as used in our main paper, are above 0.9 ($c_{80,90}$=0.96, $c_{90,100}$=0.91, $c_{100,80}$=0.90).

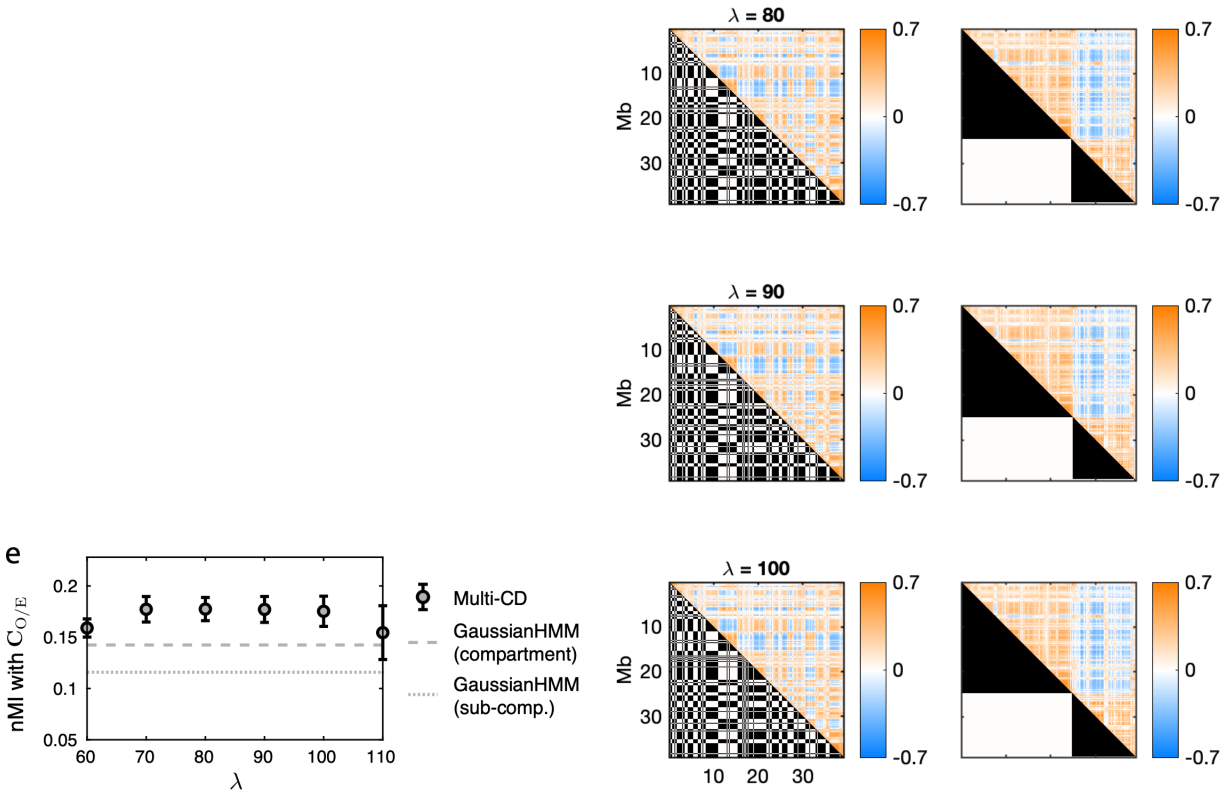

Fig R2. Comparison of compartment solutions near λ=90. Left: Reproduction of Fig 4e in the manuscript, for convenience. Right: The three rows show the three solutions at λ=80, 90 and 100 respectively. The second row (for λ=90) reproduces Fig 4b-c in the manuscript. The compartment solution is robustly recovered at the three values of λ.

As for the last part of the question, we would like to clarify that Figures 3 and 4 in our manuscript cover two different families of domain solutions, *TAD-like* (Fig 3) and *compartment-like* (Fig 4). We showed that the compartment-like solutions were only obtained as the *secondary* solution, after the most prominent correlation patterns have been explained away by the *primary* or the TAD-like solution (also see our Methods section "Incorporating the secondary group to the group model"). For the primary solution, domains at $\lambda > 70$ were uninteresting (and therefore not included in Fig 3), in the sense that the goodness measures like the nMI (Fig 3g) or the cell-to-cell similarity (Fig 3h) peaked at a smaller λ and then decreased. However, once the TAD-like solutions are removed (approximated by the diagonal band removal, Fig 4a), the remaining patterns are best captured at λ=90.

R1-3. The analysis of the link between chromatin organization and gene expression is very interesting and meaningful. The authors mentioned that the domain conservation was strongest at λ=10. Is this value for the results of GM12878 chr10, or the other four cell lines have the same value? In addition, can the authors add the analysis of two other gene expressions that support their results (Fig. 3k)?

Thanks for sharing our excitement about the analysis! The strength of domain conservation was defined across all five cell types, and therefore is not specific to any one cell type. More specifically, in Fig 3h, we calculate the cell-to-cell similarity for each pair of distinct cell types (because we analyze 5 cell types, there are 10 such pairs), and plot the average over all 10 pairs. Also see our Methods section for more details. As for the chromosome, all results shown in the main text of our paper are specific to the human chr10 (results for three additional chromosomes are shown in Fig S2).

Here we are showing ***two more examples*** where the different TAD organizations across the cell lines are consistent with different patterns of gene expressions, supporting the link between the domain structure (our TAD solutions) and the cell-type-specific gene expression (RNA-seq). In these examples, differences in the gene expression are linked to the different patterns of TAD organization in the genomic range within which the regulatory elements are located. See Fig R3 below --- we also included this figure as the new Fig S5 in the revised manuscript.

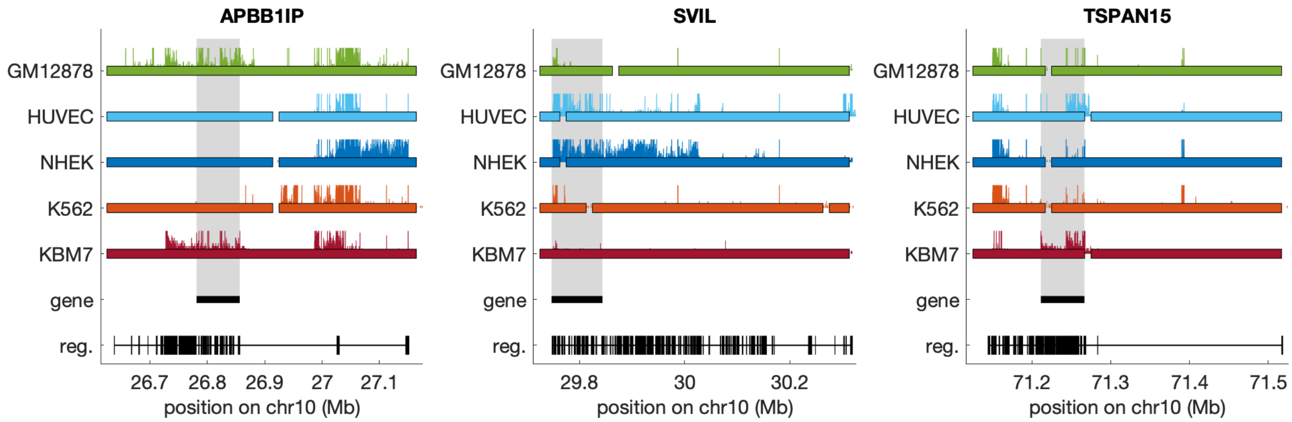

Fig R3. Three example genes: APBB1IP (example in the original manuscript); SVIL and TSPAN15 (two additional cases). Figure layout roughly replicates Fig 3k in the manuscript. Top five rows: Colored blocks indicate the TAD solutions for the five cell lines, as identified from our Multi-CD algorithm. Colored hairy lines show the RNA-seq signal for the respective cell lines. Black horizontal bar & gray shade: known position range of the gene on chromosome. Bottom row: positions of all known regulatory elements for the gene, as in the GeneHancer database (Fishilevich et al., 2007). All information is with respect to the human genome assembly GRCh37 (hg19), consistent with what we used in the manuscript.

R1-4. Only part of the short arm of chr10 is shown in this study. Did the authors construct the model for the whole chr10 and calculate the properties, or for only a part of chr10? The component of the model is not so clearly described in this study. I think the results of parameter λ will change a lot for different cells, chromosomes, and regions (Fig. S2).

As the reviewer noticed, only a 10-Mb subset of chr10 is shown in Fig 3 and Fig S2 of our manuscript. But, this is just a choice for the clarity of visualization, so that the smaller-scale features like the sub-TADs are better identified to the eyes. **The actual statistical analysis was carried out for the entire chr10.**

More specifically, we divided the chromosome chr10 (with a total length ~133 Mb) into three subsets of lengths ~40 Mb (or ~800 bins at 50-kb resolution), and applied Multi-CD separately to each of these three subsets. This subset approach helps the handling of the computational tradeoff, because the computation cost increases with the size of the genomic range covered. We confirmed that the resulting domain solutions are not sensitive to the size of the genomic subset to which the Multi-CD algorithm is applied, as long as the domains are fully included in the selected subset (see Fig C in our S3 Appendix). This means that, if there is a “true” domain that occupies a range of genomic locations 22-23Mb, our Multi-CD algorithm (at a suitable value of λ) will recover this domain no matter whether we run the algorithm on the entire 0-120Mb chromosome, a 0-40Mb subset, or a 20-30Mb subset.

Finally, we would like to clarify that λ is an auxiliary parameter in our model --- it is used like a tuning knob for the optimization problem, such that the user can scan a range of λ values and find the point where the resulting solution is maximally relevant. Here the notion of “relevant” may vary depending on the domain of interest: in order to find the TADs, we looked for the λ where the domain solutions are most strongly conserved across different cell types (Fig 3h). On the other hand, to find the compartments, we looked for the λ where the off-diagonal correlation pattern is best captured by the domain solution (Fig 4e). The exact value of this λ does not carry much information, and (as the reviewer correctly points out) the actual value of λ may depend on the specific dataset and/or the type of preprocessing.

Reviewer #2

The authors developed a Hi-C data analysis pipeline, called Multi-CD, including two steps: pre-processing and inference based on a polymer physics approach and a statistical physics sampling approach. Interestingly, they formulated the inference problem with a single parameter λ relating to multi-scale chromatin domains and tried to provide a unified framework into the hierarchical chromatin organization. Significantly, the idea of combining the correlation matrix and group modeling is very original and innovative in the field of 3D genome physical biology. As shown in Figure 6, the outcomes by this method are consistent with other reported methods. Besides, the hierarchical organization of chromatin domain families in Figure 5 clearly shows that their unified framework works well. However, this manuscript includes little biological insights. The reviewer suggests that this methodological framework is worth publishing as not a research paper but a method one. Before a final assessment, the authors need to address the following comments in a revised manuscript.

Thank you for highlighting the ideas and innovations in our method! Thank you also for the careful review of the manuscript. Regarding your comment on the scope: whereas the primary contribution of our work is a methodological framework to characterize a family of chromatin domain solutions across multiple scales, we also applied the method to analyze chromosomes from five different cell lines, which revealed a number of new insights. We believe PLoS Comp. Biol. is the best place for publishing this work.

Let us provide point-to-point responses to your comments below.

Major:

R2-1) at l399: γij has a physical unit [m^-2]. Therefore, stiffness or the spring constant is not the correct expression.

The γ_{ij} ($\sim k/k_BT$) amounts to the ``stiffness'' or the ``spring constant'' of the harmonic potential, divided by a factor of the energy unit k_BT. In the revised manuscript, we updated the text description to clarify this.

R2-2) at l441: It is not clear how to solve Equation (4) from pij to γij in this method, although the uniqueness might be confirmed.

We numerically "invert" the function p = f(γ), where $p=p_{ij}$ and $\gamma=\gamma_{ij}$ for each pair of genomic segments (i,j), by performing a simple table-search (also see Fig R4 below). The p-γ relationship is written as an integral

$$p = \int_0^{r_c} dr\, P(r;\, \gamma), \quad P(r, \gamma) = \frac{4}{\sqrt{\pi}} \gamma^{3/2} r^2 exp(-\gamma r^2)$$

where $r = r_{ij}$ is the distance between the two segments, and r_c is a cutoff distance below which the two segments can be thought of as being in contact. The integrand P(r; γ) is also given in Eq 2 in the manuscript. For the numerical procedure, we simplify this by introducing an auxiliary variable t such that $t^2 = \gamma * r^2$. With $dt = \sqrt{\gamma}\, dr$, the relationship is then rewritten as:

$$p = \int_0^{T} dt\, y(t), \quad y(t) = \sqrt{\frac{2}{\pi}}\, t^2 exp(-\frac{1}{2} t^2)$$

Note that the new upper bound T is a monotonic function of γ (related as $T^2 = \gamma * r_c^2$).

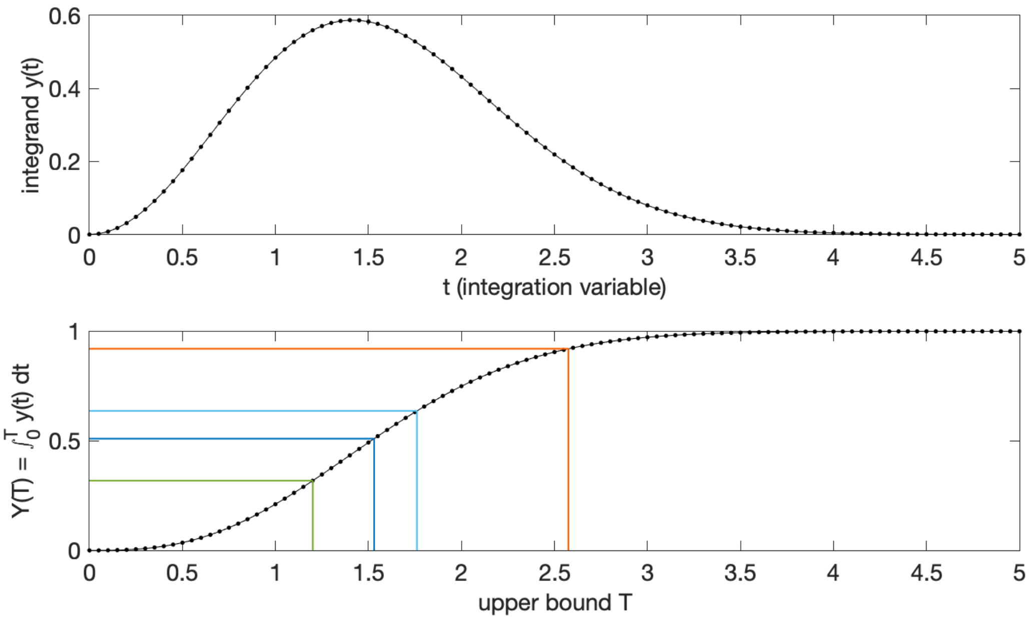

Fig R4. The top panel shows the integrand, y(t) = sqrt(2/pi) *(t^2) exp(-(1/2) t^2), as a function of the integration variable t. The bottom panel shows the definite integral of y(t) from t=0 to t=T, as a function Y(T) of the upper bound of the integral T. For the numerical integration, we used a simple rectangular rule with left-hand points, using a bin size of dt=0.05. Because the integrand is smooth and positive, this was sufficient to obtain the precision of the numerical integration that we need for the current purposes (i.e., for processing the example Hi-C data analyzed in our paper).

Because Y(T) is a smooth and monotonically increasing function of T, we can easily go from p = Y(T) to the auxiliary variable T (see the colored lines in Fig R4), then convert it back to γ at fixed r_c. It turns out that the choice of this cutoff distance r_c does not affect the correlation matrix that results from our preprocessing pipeline (this depends on an assumption that we make to handle the missing self-contact information; for details please see the text around Eq 5 in our manuscript).

This numerical solution is clearly demonstrated in our code (which is already made publicly available). We also added more description to the Methods section in the revised manuscript.

R2-3) at l444 and Equation (5): the assumption σii = σjj = σc = median(1/4γij) is too arbitrary. The reviewer guesses that the authors could not derive the one-to-one connection between γij and σij.
According to a rigorous theory of the Gaussian polymer network [55 and https://doi.org/10.1016/j.csbj.2020.08.014], the Kirchhoff or Laplacian matrix is positive semidefinite, and the smallest eigenvalue is 0 with the eigenvector proportional to (1, 1, ..., 1). Therefore, an inverse matrix does not exist. On the other hand, γij^-1 = 2(σii + σjj - 2σij) at l405 is a rigorous relationship. Besides, the matrix Σ=(σij) satisfies Σ (1, 1, ..., 1)^T = 0. Using this relation, we can derive (γij^{-1}) (1, 1, ..., 1)^T = 2 A (σ11, σ22, ..., σNN)^T, where the diagonal elements of the matrix A are N+1 and the non-diagonal elements are 1. The inverse matrix of A is \frac{1}{2N^2} B, where the diagonal elements of the matrix B are 2N-1 and the non-diagonal elements are -1. Therefore, we can derive the diagonal elements (σ11, σ22, ..., σNN) only from the matrix (γij^{-1}) and solve the equation γij^-1 = 2(σii + σjj - 2σij) without the assumption σii = σjj = σc = median(1/4γij).

We agree with the reviewer that the choice of σii = σjj = σc = median(1/4γij) is arbitrary, and thank the reviewer for working out their suggestion so clearly. However, even with this derivation, there was a reason why we needed this extra assumption in the model --- the problem is underdetermined because we do not have the diagonals γii, because the diagonal elements in the raw Hi-C matrix are missing. Whereas Hi-C reports the ligation counts for all distinct genomic segment pairs, not all experiments report the self-ligation counts, which correspond to the diagonal elements in the matrix; the dataset we primarily used for this study (Rao et al., 2014) also lacks the self-ligation information.

Therefore, we needed to make *some* assumptions to fill in the missing information for the diagonal elements. We tried to make this as transparent as possible in the description of the methods. Our choice of using the median value is not particularly justifiable, but it has an advantage that the resulting correlation matrix becomes insensitive to the choice of the cutoff distance r_c, and it seems to work in a stable way. We will be happy to see a future work that improves this part of the model.

R2-4) Although the reviewer cannot verify the Matlab codes, information regarding the computational calculation time to obtain an optimal solution for a parameter λ must be useful for PLOS CB readers.

Our original manuscript had a brief discussion of the issues related to the computational cost as part of S3 Appendix, but we agree with the reviewer that this information should be made more accessible to be useful. We added a subsection that discusses the parameters in the model that affects the computational cost, along with some practical notes to guide use cases, under a new heading "Computational cost" in S3 Appendix. The documentation to our Matlab code also includes a similar guide.

R2-5) In terms of bioinformatics and computational approaches, the parameter λ would be an excellent measure to identify multi-scale chromatin domains. Also, the authors have discussed the interpretation of λ as the negative chemical potential. However, if the authors are responsible for the meaning of λ in terms of physics, they should discuss and propose a way to find a physical meaning of λ by experiments.

Our interpretation of the parameter λ as the (negative) effective chemical potential highlights the mathematical analogy to the well-studied statistical physics formulation, which provides a better understanding of the problem at hand and guides the inference approach. In terms of our clustering problem, the effective chemical potential amounts to the difference in the effective Hamiltonian that is associated with the creation of a new domain or the merging of two domains into one. Apparently, the chromatin domains are not real particles, and therefore they do not have any true physical chemical potential; that is why we choose to treat λ as a variable parameter and attempt to find the value of λ that is most meaningful to the specific context of the problem.

Minor:

R2-i) typo at l76: the presence "of" of cell-to-cell ... Remove "of."

Thanks, the typo has been fixed.

R2-ii) at l396: Equation (2) does not represent the distance distribution but the probability density function of the distance.

Thank you for the careful reading. We corrected this in the revised manuscript.
