# Supplementary material for: A unified framework for inferring the multi-scale organization of chromatin domains from Hi-C
Source: PLoS Comput Biol. 2021 Mar 16;17(3):e1008834. doi: 10.1371/journal.pcbi.1008834 (PMC7997044; doi:10.1371/journal.pcbi.1008834)
Supplement: Attachment — Submitted filename: Response.docx [file pcbi.1008834.s014.docx]

Response to reviewers (reviewer 2)

We present the reviewer comments in **black** and our responses in **blue**.

**Comment 1 to the reply R2-3:**

I understood that the authors used the tar formatted Hi-C matrix data, and the diagonal elements were missing in the format.

But, if one uses the hic formatted data, the diagonal elements are available. Moreover, invalid pairs, including self-ligation, are eliminated in a Hi-C analysis pipeline from FASTQ data. Therefore, the diagonal element p_ii indicates not self-ligation counts but the contact frequency within the i-th genomic region.

I am looking forward to your future work with improving these parts.

As the referee has correctly pointed out, depending on the Hi-C pipeline, the Hi-C-derived contact matrix p_ij_ may or may not contain the diagonal entries, p_ii_, which reflect the contact frequency within each genomic region. In this work, we treat each genomic region as a source of information (or a point-like monomer) and use the correlation between the different regions as the input data for Multi-CD analysis. Please note, however, that the ***correlation*** of self-interaction is unity by definition ((C)_ii_ = 1), regardless of the value of p_ii_ or covariance σ_ii_ from Hi-C-derived matrix.

By introducing a quantity σ_c_ as the median value of 1/4γ_ij_’s, we are trying to provide a simple way to ensure that the resulting correlation matrix (C) has balanced fractions of positive and negative correlation, making the most of the value range [−1, 1] such that the global correlation pattern is clearly visible. We updated the Methods section of our revised manuscript to better discuss these issues.

**Comment 2 to the reply R2-5:**

The authors should add a discussion on the physical implication of λ.

We added this paragraph near the beginning of the Results section in the manuscript, where we outline our approaches and methods:

**(L117)** There is a mathematical analogy between the form of the objective function in Eq.1, and the grand canonical ensemble in statistical physics (see Methods). The analogy to the well-studied physical formulation provides a useful conceptual framework, and justifies the use of efficient inference procedures such as simulated annealing (see Methods). We also note that, in this view, the parameter λ has a physical interpretation as the effective *chemical potential* of a domain in the solution, which is associated with the creation of a new domain or the merging of two domains into one.

Meanwhile, the Methods section already provides an explanation on how the domain structure is identified when the λ is tuned to a larger or smaller value. For convenience, we also copy the corresponding text here:

“The Lagrange multiplier λ (≥ 0) is a parameter that controls how strongly the problem prefers parsimonious solutions: with a larger λ, the solution s∗ for the optimization problem would tend to have fewer domains. Equivalently, a larger λ prefers a larger-scale domain solution. Solving at multiple values of λ, therefore, may reveal the multi-scale domain structure in data.”
